# Docetaxel and cisplatin induction chemotherapy with or without fluorouracil in locoregionally advanced head and neck squamous cell carcinoma: A real-world data study

**DOI:** 10.1016/j.bjorl.2025.101572

**Published:** 2025-02-28

**Authors:** Matheus Yung Perin, Vivian Naomi Horita, Daniel Naves Araújo Teixeira, Joyce Gruenwaldt, Eduardo Baldon Pereira, Carlos Takahiro Chone, Gustavo Jacob Lourenço, Ligia Traldi Macedo, Carmen Silvia Passos Lima

**Affiliations:** a Faculdade de Ciências Médicas da Universidade Estadual de Campinas, Departamento de Radiologia e Oncologia, Serviço de Oncologia Clínica, Campinas, SP, Brazil; b Faculdade de Ciências Médicas da Universidade Estadual de Campinas, Departamento de Oftalmologia e Otorrinolaringologia, Campinas, SP, Brazil; c Faculdade de Ciências Médicas da Universidade Estadual de Campinas, Departamento de Radiologia e Oncologia, Serviço de Radioterapia, Campinas, SP, Brazil; d Faculdade de Ciências Médicas da Universidade Estadual de Campinas, Laboratório de Genética do Câncer, Campinas, SP, Brazil

**Keywords:** Head and neck squamous cell carcinoma, Induction therapy, Response rate, Toxicity, Survival

## Abstract

**Objective:**

To evaluate outcomes of locoregionally advanced patients with Head and Neck Squamous Cell Carcinoma (HNSCC) treated with Induction Therapy (ICT).

**Methods:**

Toxicity, response rate, and Event-Free Survival (EFS) and Overall Survival (OS) were evaluated in patients treated with docetaxel, cisplatin, and 5-Fluorouracil (TPF) or docetaxel and Cisplatin (TP).

**Results:**

ICT regimens did not alter response to ICT, and patients’ EFS and OS. Cox multivariate analysis identified stable or progressive disease (HR = 5.56) and interval between cycles ≥28 days (HR = 2.79) as predictors of lower EFS, and ECOG ≥ 1 (HR = 3.42), stable or progressive disease (HR = 4.67), and interval between cycles ≥28 days (HR = 2.73) as predictors of lower OS.

**Conclusion:**

Our findings indicate TP as a good treatment option for locoregionally advanced HNSCC, especially in socioeconomically limited settings.

**Level of evidence:**

3.

## Highlights

• Induction therapy with TPF or TP has been indicated for advanced HNSCC patients.
• TPF is more toxic and requires infusion device or in-hospital administration of 5-FU.
• We analyzed the outcomes of advanced HNSCC patients treated with TPF or TP.
• ICT regimens did not alter patients’ toxicity, response rate and survival.
• TP is a good treatment option for advanced HNSCC in socioeconomically limited settings.

## Introduction

Head and Neck Squamous Cell Carcinoma (HNSCC) is the 7th most common cancer worldwide, with 878,348 new cases and 364,339 deaths reported annually.^1^ In Brazil, the National Cancer Institute (INCA) estimated 23,000 new cases and 11,000 deaths each year during the period of 2023–2025, making it the 8th most common tumor.^2^

The treatment of patients with HNSCC varies based on primary site and tumor staging. Surgical Resection (SR) or Radiotherapy (RT), often accompanied by Chemotherapy (CT), are treatment options for patients with localized tumors.^3^ Patients with unresectable or metastatic tumors undergo palliative CT, preferably with platinum-based regimens.^4^ Approximately 60% of HNSCC patients are diagnosed at a locally or locoregionally advanced stage^5^, ^6^ for which a multimodal approach is necessary.^7^ In this context, patients with resectable tumors undergo SR with or without adjuvant RT or Chemoradiotherapy (CTRT). Conversely, patients not amenable to SR receive CTRT as definitive treatment, or Induction Chemotherapy (ICT) followed or not by CTR.^3^, ^8^, ^9^, ^10^, ^11^, ^12^, ^13^

In the last scenery, docetaxel plus Cisplatin (TP^10^, ^14^ or TP followed by CTRT^15^ were first described as an effective ICT with acceptable safety profile for advanced, recurrent or metastatic HNSCC in Phase 2 studies. In Phase 3 studies, taxane (paclitaxel or docetaxel), cisplatin, and 5-Fluorouracil (TPF) followed by CTRT was superior to Cisplatin plus 5-Fluorouracil (CF) followed by CTRT in response to treatment,^16^, ^17^ Overall Survival (OS^18^ and Progression-Free (PFS) and OS^11^, ^19^ of patients with locally advanced and unresectable HNSCC; higher response to treatment, loco-regional control, PFS and OS were also seen in patients treated with TPF followed by cisplatin or cetuximab plus RT compared to those treated with TPF followed by the concomitant treatment.^12^ In contrast, no differences in survival were seen in patients treated with TPF followed by CTRT and patients treated with CTRT alone.^20^ The superiority of TPF over CF in locoregional and distant tumor control was evidenced in previous meta-analysis,^21^ phase 3 trial^17^ and non-systematically review.^22^ As expected, TPF was also compared with TP in patients with borderline resectable oral SCC in a single phase 3 study, and a survival benefit was attributed to TPF when compared to TP.^23^, ^24^

Despite the superiority of TPF over TP in response rate, loco-regional control, and survival of patients with advanced HNSCC seen in a single study, unequivocal disadvantages have been attributed to the regimen, as grade 3 or above adverse events (neutropenia),^13^, ^24^ and the need of infusion devices or inpatient beds for continuous 5-fluorouracil infusion, which clearly increases the costs of treatment.^23^ Additionally, social factors affecting HNSCC patients may limit its application in certain areas of current clinical practice.

The current study aimed to analyze patients with locoregionally advanced HNSCC treated with TPF or TP followed by CTRT at the public General Hospital of the University of Campinas with the purpose of developing an ICT protocol applicable to services with limited resources.

## Methods

### Participants

In this retrospective study, patients with locoregionally advanced HNSCC, tumor at stage III or IVA-B (T4 and/or N2b, N2c or N3), diagnosed at the General Hospital of the University of Campinas, and treated with ICT using TPF or TP from January 14th, 2015, to November 24th, 2021, were included.

Clinicopathological information, such as age at diagnosis, gender, ECOG, Body Mass Index (BMI), smoking status, alcohol consumption, and comorbidities, as well as tumor information, including site, histological grade, Human Papillomavirus (HPV) status, and stage, were obtained from medical records. Patients were classified as never, former, or current smokers,^25^ as drinkers or non-drinkers,^26^ and with reduced weight, normal weight, overweight, and obese.^27^ The diagnosis of HNSCC and the histological tumor grade were based on World Health Organization criteria.^28^ Tumor stage was identified according to the American Joint Committee on Cancer (AJCC) criteria,^29^ and HPV status was determined by immunohistochemistry through the presence of p16.^30^

### Treatment protocol

As this was a retrospective study, the decision regarding the ICT regimen was not made by the investigators but reflected real-world clinical practice. The choice between TPF and TP as Induction Chemotherapy (ICT) was based on the clinical judgment of the responsible oncologist, considering patient-specific factors such as performance status, comorbidities, and tolerance to intensive regimens. Additionally, the availability of a hospital bed for the continuous intravenous infusion of 5-fluorouracil was a practical determinant. All patients received anti-emetic prophylaxis with intravenous ondansetron and dexamethasone pre-infusion, in addition to oral ondansetron 8 mg and dexamethasone 4 mg twice daily for the following three days.^31^ Mannitol and hydration with saline solution, potassium chloride, and magnesium sulfate were administered as previously reported.^32^

The TPF protocol consisted of three cycles at 21-day intervals, with intravenous docetaxel 75 mg/m^2^ and cisplatin 100 mg/m^2^ on D1 if the Glomerular Filtration Rate (GFR) was less than 60 mL/min, and continuous intravenous 5-fluorouracil 1000 mg/m^2^ on days 1‒4.^19^ The TP protocol included three cycles of intravenous administration of docetaxel 75 mg/m^2^ and cisplatin 75 mg/m^2^ on D1, repeated every 3 weeks if the GFR was greater than 60 mL/min.^33^ Dose reductions were implemented in cases of advanced age or low performance status. Additionally, reductions in doses of chemotherapeutic agents during treatment were considered in the presence of grade 3 or above toxicity, with the first reduction being 20% and the second, an additional 20%.

ICT toxicity was graded according to the Common Terminology Criteria for Adverse Events (CTCAE) version 5.0.^34^ The Response Rate (RR) to ICT was classified as Complete Response (CR), Partial Response (PR), Stable Disease (SD), or Progression of Disease (PD) using the Response Evaluation Criteria in Solid Tumors (RECIST) guidelines version 1.1.^35^

Patients were assigned to receive CTRT beginning 3–8 weeks after the end of the third cycle of ICT (day 22 to day 56 of cycle 3). Weekly cisplatin at 40 mg/m^2^ or carboplatin at an Area Under the Curve (AUC) of 2 was given for a maximum of 7 doses during RT. The dose administered to the primary tumor was 70 Gy (2 Gy per day, 5 days per week). Uninvolved lymph nodes were treated with 50 Gy and involved lymph nodes received 60–70 Gy. All patients were treated using a 2-field or 3-field technique.

After the end of treatment, patients were seen at outpatient appointments every three months for two years, then every six months as per routine care. Imaging exams and laryngoscopy were performed periodically and whenever necessary. Patients who failed to respond to ICT followed by CTRT or relapsed during follow-up received palliative CT.

The induction regimen, number of cycles, interval between induction cycles (<28 days or ≥28 days), toxicity events with grades equal to or above 3, and types of response to ICT were recorded for all enrolled patients.

### Statistics analysis

All data were deposited on the Research Electronic Data Capture (RedCap) platform.^36^, ^37^ Descriptive analyses were performed according to variable distributions. Wilcoxon signed-rank test and Fisher exact tests were used to verify the balance between groups according to clinicopathological characteristics. Dates of diagnosis, disease progression, death, and last follow-up were obtained from medical records. Event-Free Survival (EFS) and OS were defined as the period from the date of diagnosis to the occurrence of progression, recurrence, the last appointment, or death due to the tumor, and from the diagnosis to the last appointment or death from any cause, respectively. EFS and OS were assessed using the Kaplan-Meier method, and the log-rank test was applied to verify significant differences in the curves. The impact of clinicopathological characteristics on patients’ survival was assessed through univariate and multivariate Cox regression. All variables with a *p*-value < 0.10 in the univariate were subjected to multivariate analysis using a stepwise approach.

All statistical analyses were conducted using RStudio (version 2023.06.1) and we considered differences statistically significant when the p-values were less than 0.05.

### Ethics aspects

This study received ethical approval from the Ethical Committee (CAAE 22425319.6.0000.5404). Due to the nature of study, which involved patients who had passed away, our protocol included a waiver of informed consent form.

## Results

### Patient’s clinicopathological aspects

**Fig. 1:**
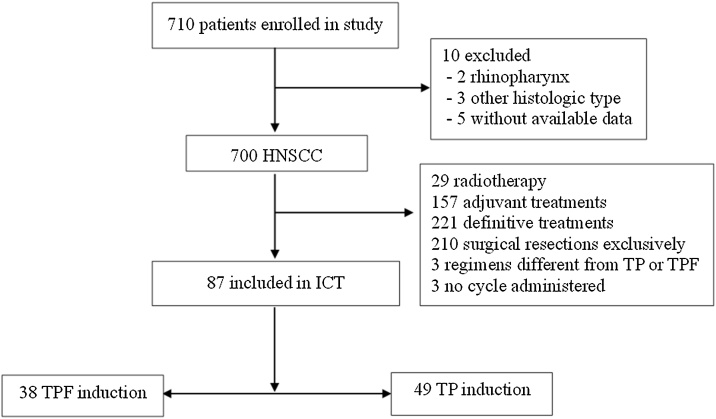
CONSORT diagram for patient selection. HNSCC, Head and Neck Squamous Cell Carcinoma; ICT, Induction Chemotherapy; TPF, Taxane, Platin, and 5-Fluorouracil; TP, Taxane and Platin.

Eighty-seven out of 710 patients with HNSCC seen during the period of study were treated with ICT, being 38 with TPF and 49 with TP (Fig. 1).

The clinicopathological aspects of the total group of patients (n = 87) and of patients stratified by ICT protocol are presented in Table 1. The mean age of the total group of patients was 56 years. Most patients were males, smokers or former smokers, drinkers or former drinkers, with ECOG status 0 or I and reduced or normal BMI. Half of the tumors were in pharynx and the remaining tumors were equally distributed in oral cavity and larynx. Tumors were mainly moderately differentiated and at stage IV. Nearly half of patients experienced toxicity of grade 3 or greater, with neutropenia being the more common toxicity, there were no treatment-related death. Most patients achieved CR or PR, and near one-third of patients initiated the second ICT cycle after a 28-day interval. An excess of ECOG 0 or 1 was seen in TPF group and an excess of males in TP group, but no significant differences in clinicopathological aspects, as age, smoking and alcohol intake, BMI, tumor location, grade and TNM stage, toxicities grade 3 or above, treatment response, and cycles interval, were seen in patients treated with TPF and TP.

**Table 1:** Clinicopathological characteristics of 87 patients with head and neck squamous cell carcinoma treated with induction protocols.

Variable	Total (N = 87), n (%)	TPF group (N = 38), n (%)	TP group (N = 49), n (%)	p-value
Age at diagnosis				
Mean (± SD)	56 (± 9.3)	55 (± 10.9)	57 (± 7.8)	0.34
≤56 years	39 (44.8)	18 (47.4)	21 (42.9)	0.83
>56 years	48 (55.2)	20 (52.6)	28 (57.1)	
**Sex**				
Female	9 (10.3)	7 (18.4)	2 (4.1)	**0.03**
Male	78 (89.7)	31 (81.6)	47 (95.9)	
Smoking				
Yes	38 (43.7)	13 (34.2)	25 (51.0)	0.09
Former	41 (47.1)	19 (50.0)	22 (44.9)	
Never	8 (9.2)	6 (15.8)	2 (4.1)	
Alcohol intake				
Yes	31 (35.6)	12 (31.6)	19 (38.8)	0.08
Former	49 (56.3)	20 (52.6)	29 (59.2)	
Never	7 (8.1)	6 (15.8)	1 (2.0)	
ECOG^a^				
0	33 (38.9)	9 (24.3)	24 (50.0)	**0.02**
1	45 (52.9)	26 (70.3)	19 (39.6)	
2	7 (8.2)	2 (5.4)	5 (10.4)	
BMI				
Underweight	27 (31.0)	14 (36.8)	13 (26.5)	0.48
Healthy weight	39 (44.8)	15 (39.5)	24 (49.0)	
Overweight	16 (18.4)	8 (21.1)	8 (16.3)	
Obesity	5 (5.8)	1 (2.6)	4 (8.2)	
Tumor location				
Oral cavity	21 (24.2)	9 (23.7)	12 (24.5)	0.95
Oropharynx	45 (51.7)	19 (50.0)	26 (53.1)	
Larynx	21 (24.1)	10 (26.3)	11 (22.4)	
Grade				
Well differentiated	5 (5.8)	2 (5.3)	3 (6.1)	0.76
Moderately differentiated	61 (70.1)	30 (78.9)	31 (63.3)	
Poorly differentiated	11 (12.6)	4 (10.5)	7 (14.3)	
Not available	10 (11.5)	2 (5.3)	8 (16.3)	
HPV16 status^a^				
Positive	9 (36.0)	3 (42.9)	6 (33.3)	0.67
Negative	16 (64.0)	4 (57.1)	12 (66.7)	
T category				
T1	3 (3.4)	1 (2.6)	2 (4.1)	0.79
T2	5 (5.7)	2 (5.3)	3 (6.1)	
T3	21 (24.1)	10 (26.3)	11 (22.4)	
T4	58 (66.8)	25 (65.8)	33 (67.3)	
N category				
N0	3 (3.4)	1 (2.6)	2 (4.1)	1.00
N1	5 (5.7)	2 (5.3)	3 (6.1)	
N2	36 (41.4)	16 (42.1)	20 (40.8)	
N3	43 (49.5)	19 (50.0)	24 (49.0)	
TNM stage				
III	4 (4.6)	2 (5.3)	2 (4.1)	1.00
IV	83 (95.4)	36 (94.7)	47 (95.9)	
Toxicity grade 3 or more				
No	47 (54.0)	19 (50.0)	28 (57.1)	0.52
Yes	40 (46.0)	19 (50.0)	21 (42.9)	
Treatment response^a^				
Complete	15 (19.2)	5 (15.2)	10 (22.2)	0.28
Partial	47 (60.3)	18 (54.4)	29 (64.4)	
Stable disease (SD)	9 (11.5)	5 (15.2)	4 (8.9)	
Disease progression (DP)	7 (9.0)	5 (15.2)	2 (4.5)	
Complete or partial	62 (79.5)	23 (69.7)	39 (86.7)	0.09
SD or DP	16 (20.5)	10 (30.3)	6 (13.3)	
Cycles interval^a^				
<28 days	50 (67.6)	17 (56.7)	33 (75.0)	0.16
≥28 days	24 (27.6)	13 (43.3)	11 (25.0)	

N, Number; TPF, Taxane, Platin, and Fluorouracil; TP, Taxane and Platin; SD, Standard Deviation; ECOG, Eastern Cooperative Oncology Group performance status; BMI, Body Mass Index; TNM, Tumor-lymph Node-Metastasis.

a The number of patients differed from the initial one because it was not possible to obtain pertinent information in all cases. Cycles interval: mean interval between 1st and 2nd cycles of induction chemotherapy. Bold numbers represent significant values (*p* < 0.05).

### Patients’ survival

The median follow-up time was 22.6 months (range: 1.2–93.8 months). At to the last survival analysis (April 22nd, 2024), 49 patients had died (46 from the disease effects and three from other causes) and 38 patients were alive (14 with the disease and 24 disease-free), while 39 patients experienced a tumor relapse.

**Fig. 2:**
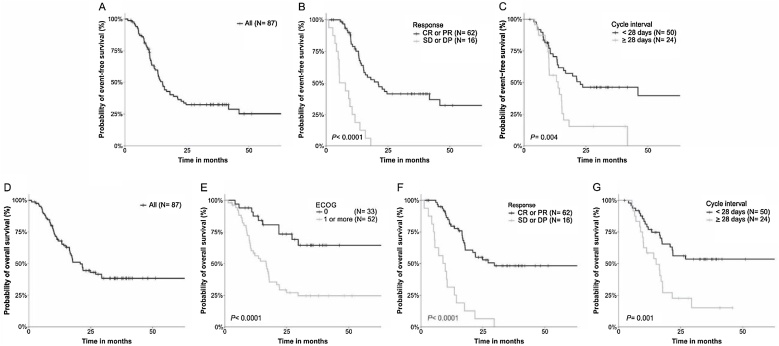
Survival curves according to Event Free Survival (EFS) and Overall Survival (OS) in head and neck squamous cell carcinoma treated with Induction Therapy (ICT). (A) EFS in general population. (B) EFS was lower in patients with Stable Disease (SD) or Progressive Disease (PD) than in those with Complete Response (CR) or Partial Response (PR) after ICT. (C) EFS was lower in patients with interval between ICT cycles equal or above 28 days. (D) OS in geral population. (E) OS was lower in patients with Eastern Cooperative Oncology Group (ECOG) performance status equal or above 1. (F) OS was lower in patients with SD or PD after ICT. (G) OS was shorter in patients with interval between ICT cycles equal or above 28 days (Kaplan-Meier estimates).

The two-year and five-year EFS rates of patients of the total group were 33.8% and 25.3%, respectively (Fig. 2A). At 24 months of follow-up, EFS was lower in patients with ECOG equal or above 1 (18.3% vs. 58.4%, *p* = 0.001), underweight patients (8.2% vs. 45.3%, *p* = 0.0001), patients treated with TPF (17.6% vs. 46.4%, *p* = 0.005), patients who exhibited SD or PD after ICT (0.0% vs. 43.1%, *p* < 0.0001) (Fig. 2B), and patients with interval of 28 days or more between ICT cycles (15.2% vs. 46.3%, *p* = 0.004) (Fig. 2C) when compared to others (Kaplan-Meier estimates). At 60 months, ESF in TPF was lower than in TP (14.7% vs. 31%, *p* = 0.005) (Kaplan-Meier estimates). The variables remained predictors of shorter EFS in univariate Cox analysis. Multivariate Cox multivariate analysis showed that patients who exhibited SD or PD, and those experiencing long interval between cycles had 5.56 and 2.79 more chances of presenting tumor progression/recurrence or death from disease than others, respectively (Table 2).

**Table 2:** Cox regression analysis examining the association of clinical and tumor characteristics in 87 patients with head and neck squamous cell carcinoma.

Variable	Total N	Event-free survival					Overall survival				
		N of event	Univariate analysis		Multivariate analysis		N of event	Univariate analysis		Multivariate analysis	
			HR (95% CI)	p-value	HR (95% CI)	p-value		HR (95% CI)	p-value	HR (95% CI)	p-value
Age											
≤56 years	39	27	Reference	0.56	NC	NC	22	Reference	0.95	NC	NC
>56 years	48	29	1.16 (0.50–1.45)				27	1.01 (0.57–1.78)			
Sex											
Female	9	6	1.75 (0.24–1.33)	0.19	NC	NC	8	2.10 (0.20–1.12)	0.08	NC	NC
Male	78	50	Reference				43	Reference			
ECOG^a^											
0	33	16	Reference	0.001	Reference	0.12	10	Reference	0.0004	Reference	**0.005**
1 or more	52	38	2.59 (1.43–4.68)		1.96 (0.83–4.62)		37	3.48 (1.72–7.04)		3.42 (1.44–8.10)	
BMI											
Underweight	27	23	2.37 (1.37–4.13)	0.002	1.41 (0.32–1.54)	0.38	20	1.87 (1.05–3.33)	0.03	1.05 (0.42–2.09)	0.88
Others	60	33	Reference		Reference		29	Reference		Reference	
Tumor location											
Oral cavity	21	16	1.11 (0.60–1.32)	0.57	NC	NC	15	1.26 (0.52–1.18)	0.25	NC	NC
Oropharynx	45	26	Reference				22	Reference			
Larynx	21	14					12				
Tumor grade											
Well	5	3	1.17 (0.47–1.50)	0.57	NC	NC	2	1.17 (0.44–1.60)	0.61	NC	NC
Moderately	61	41	Reference				36	Reference			
Poorly	11	7					6				
TNM stage											
III	4	3	1.03 (0.30–3.09)	0.95	NC	NC	3	1.22 (0.25–2.62)	0.73	NC	NC
IV	83	53	Reference				46	Reference			
ICT											
TPF	38	30	2.08 (1.22–3.54)	0.006	1.49 (0.32–1.37)	0.27	26	1.82 (1.03–3.21)	0.03	1.17 (0.39–1.80)	0.66
TP	49	26	Reference		Reference		23	Reference			
Toxicity											
Some	40	25	Reference	0.58	NC	NC	22	Reference	0.84	NC	NC
None	47	31	1.16 (0.68–1.96)				27	1.06 (0.60–1.86)			
Response^a^											
CR or PR	62	34	Reference	< 0.0001	Reference	**<0.0001**	28	Reference	<0.0001	Reference	**0.0002**
SD or DP	16	16	5.99 (3.15–11.39)		5.56 (2.49–12.41)		16	5.48 (2.89–10.38)		4.67 (2.03–10.77)	
Cycles interval											
<28 days	50	26	Reference	0.005	Reference	**0.002**	21	Reference	0.002	Reference	**0.005**
≥28 days	24	19	2.36 (1.28–4.35)		2.79 (1.43–5.41)		19	2.62 (1.39–4.91)		2.73 (1.35–5.53)	

N, Number; HR, Hazard Ratio; CI, Confidence Interval; ECOG, Eastern Cooperative Oncology Group performance status; BMI, Body Mass Index; Tumor grade, Well, moderately, or poorly differentiated; TNM, Tumor-lymph Node-Metastasis; ICT, Induction Chemotherapy; TPF, Taxane, Platin, and Fluorouracil; TP, Taxane and Platin. Response: CR, Complete Response; PR, Partial Response; SD, Stable Disease, and DP, Disease Progression. Cycles interval, mean interval between 1st and 2nd cycles of ICT in days. NC, Variable Not Computed in multivariate analysis.

a The number of individuals listed differs from the initial count due to the unavailability of relevant information in some cases. Bold numbers represent significant values (*p* < 0.05) following multivariate analysis.

The two-year and five-year OS rates were 44.5% and 38.3%, respectively (Fig. 2D). At 24 months of follow-up, OS was shorter in patients with ECOG equal or above 1 (29.0% vs. 73.2%, *p* < 0.0001) (Fig. 2E), underweight patients (31.1% vs. 50.6%, *p* = 0.02), patients submitted to TPF (31.3% vs. 55.3%, *p* = 0.03), patients who exhibited SD or PD (6.2% vs. 54.9%, *p* < 0.0001) (Fig. 2F), and those with interval of 28 days or more between ICT cycles (22.4% vs. 56.0%, *p* = 0.001) (Fig. 2G) (Kaplan-Meier estimates). At 60-months follow up, the OS in TPF was lower than in TP (26.8% vs. 47.6%, *p* = 0.03) (Kaplan–Meier estimates). The associations were confirmed in univariate Cox analysis. Patients with ECOG equal or above 1, who exhibited SD or PD, and with long interval between cycles of ICT had 3.42, 4.67, and 2.73 more chances of evolving to death than others, respectively, in multivariate Cox analysis (Table 2).

## Discussion

In this retrospective study, we evaluated the toxicities, tumor control, and survival outcomes of patients with locoregionally advanced HNSCC who underwent ICT with TPF, or TP followed by concurrent CTRT.

Initially, we observed similar clinicopathological characteristics between our patients and those reported in other studies worldwide,^1^ suggesting our sample’s representativeness in medical practice settings. The more consistent difference between our cases and others seemed to occur in HNSCC carcinogenesis, such as the high impact of tobacco and alcohol intake,^38^, ^39^ which was previously associated with poor prognosis.^40^, ^41^ Nevertheless, it is worth to comment that the HPV16 status was not evaluated in approximately two thirds of the sample and, and for this reason we cannot exclude the participation of the virus in association with tobacco and alcohol in the carcinogenesis of the tumor.

Secondly, we observed that nearly 50% of patients experienced Grade 3 or above toxicity, with neutropenia being the most common adverse event, 80% of patients achieved CR or PR, and 30% of patients received the second cycle of ICT after a 28-day interval. No significant differences in toxicity, response rate and interval between cycles of ICT were observed in patients treated with TPF or TP regimen.

Variable frequencies of neutropenia grade 3 or above were observed in patients treated with TP (20%–95%)^14^, ^42^ and with TPF^16^, ^17^, ^20^ in phase 2 and 3 trials, including the neutropenia rate found in our study. Neutropenia grade 3 or above was more common in patients underwent TPF-treated patients compared to those receiving TP in randomized phase 3 trials in HNSCC^24^ and nasopharyngeal carcinoma,^43^ but our study did not corroborate this finding.

Variable frequencies of CR/PR after ICT were observed in HNSCC patients treated with TP (54%–88%)^10^, ^14^, ^15^ and with TPF (65%–80%)^11^, ^17^, ^18^, ^19^ in phase 2 and 3 trials, with responses to treatment being described as more common in TPF-treated patients than in those treated with TP.^13^, ^21^, ^22^ It is important to note that we identified CR/PR to ICT in a significant proportion of cases, but no significant differences in response rates were seen in TPF and TP groups.

To our knowledge, there are no clear descriptions of ICT cycle delays in previously conducted studies, which make comparison of our results difficult. Therefore, we can point out that they were relatively common in patients treated in our service and were similar in TPF-treated and TP-treated patients.

It is possible that characteristics specific of our patients may explain the above-mentioned differences in toxicity and response to treatment among studies; but we cannot exclude that such differences could be better detected in phase 3 trials. We also report that delays in cycles resulted mainly by the disproportion between the increase in number of patients with advanced HNSCC for ICT and the expansion of the institutional clinical oncology service.

Third, it was found, using Kaplan–Meier method, that the 60-month EFS and OS rates of the total group of patients treated with ICT followed by CTRT were 25.3% and 38.3%, respectively. At the same time and unexpectedly, our patients treated with TPF had EFS and OS lower than those treated with TP (26.8% vs. 47.6%; 14.7% vs. 31%, respectively). The 60-month PFS was 15.4% vs. 22.5% and OS was 18.5% vs. 23.9% in TP and TPF arms, respectively, in a single phase 3 study conducted by Noronha et al. (2024^24^ in borderline resectable oral SCC; it was found that the TPF regimen resulted in a 5.4% absolute improvement in OS and a 23.8% proportional increment in OS over TP.

EFS and OS of our patients was slightly higher than those observed in the study conducted by Noronha et al. (2024).^24^ It is possible that the differences in survival observed are attributable to distinct numbers of patients treated (87 vs. 495 cases), study designs (retrospective vs. randomized), numbers of ICT cycles (three cycles vs. two cycles), and types of treatment after ICT (CTRT vs. SR, definitive CTRT or palliative CT). At this point in the discussion, it is essential to comment that we did not observe superiority of TPF or TP regimen in EFS and OS of patients, when confounding factors were ruled out by Cox multivariate analysis. It is also important to consider that excesses of T4b (67.3% vs. 56.3%, *p* = 0.013) and stage IVb (70.6 vs. 60.7%, *p* = 0.023) tumors were included in TP group in the study of Noronha et al., which may have acted as confounding factors in patient survival analyses.

Finally, Cox multivariate analysis identified SD or PD (HR = 5.56) and interval between cycles ≥28 days (HR = 2.79) as independent predictors of lower EFS, and ECOG ≥ 1 (HR = 3.42), stable or progressive disease (HR = 4.67), and interval between cycles ≥ 28 days (HR = 2.73) as independent predictors of lower OS.

The impact of the response to ICT in patients’ survival was not evaluated by Noronha et al. (2024).^24^ However, it is already known that HNSCC patients with achieving CR after neoadjuvant therapy had an improved survival than the remaining patients.^44^, ^45^

Noronha et al. (2024^24^ did not evaluate the impact of the delays in ICT in patients’ survival, but some trials and meta-analyses showed superiority of high-density chemotherapy regimens, different from those used as ICT, over the same regimens with conventional intervals in OS in patients with small-cell lung cancer^46^, ^47^ and breast cancer.^48^ In HNSCC, a phase 2 trial showed benefit in survival with TPF dose-dense.^49^ In addition, few trials found delay in CT as independent prognostic factor for worse outcomes in platin delays in ovarian cancer^50^ and non-small cell lung cancer,^51^ as well as in colorectal cancer independent of the regimen.^52^

Noronha et al. (2024^24^ also identified ECOG < 1 as an independent prognostic factor (HR = 1.516; *p* = 0.001) for longer survival in patients treated with TPF or TP. Additionally, longer survival was seen in patients with advanced^44^ or metastatic^53^ HNSCC with good performance compared to others.

Thus, our data present preliminary evidence that PR or DP after ICT and delays between ICT cycles alter substantially the survival of patients with locoregionally advanced HNSCC, and support ECOG < 1 as an independent predictor of longer survival in this scenery.

It is worth highlighting that Brazil is a country of great economic contrasts, with numerous hospitals that suffer from a lack of financial resources, where infusion devices or inpatient beds for continuous 5-fluorouracil infusion are used sparingly.^54^, ^55^ Numerous other developing countries have the same problems.^56^, ^57^, ^58^ Moreover, the costs of treating cancer patients are also a concern for medical service managers of developed countries, who certainly have greater financial resources, but also limited.

## Conclusion

Our findings indicate unsatisfactory clinical condition of patients and long interval between cycles of ICT as independent predictors of short survival, indicating that ICT should begin early and be administered within the recommended intervals between cycles. Our findings also indicate non-ideal response to ICT as an independent predictor of short survival, and since similar efficacy and toxicity of TPF and TP in the treatment of patients with locoregionally advanced HNSCC, TP may be the option in socioeconomically limited settings.

We acknowledge the inherent limitations of our study, such as its retrospective design, participation of a single center, and the relatively small number of patients treated with TPF and TP, which may have limited statistical power in group comparisons. Additionally, there is significant missing data regarding HPV status, a recognized prognostic factor for oropharyngeal HNSCC,^40^, ^59^, ^60^ which could act as confounding factor in our analyses of survival. More comprehensive research with a larger sample size and evaluation of HPV status is needed to confirm the findings from this study.

## Funding

The authors received no specific funding for this work.

## Declaration of competing interest

The authors declare no conflicts of interest.
